# Evolutionary game theory: lessons and limitations, a cancer perspective

**DOI:** 10.1038/sj.bjc.6605444

**Published:** 2009-11-17

**Authors:** J W McEvoy

**Affiliations:** 1 The Johns Hopkins Hospital, 600 N. Wolfe Street, Baltimore, MD 21287, USA

**Sir,**

Dingli *et al* report the use of evolutionary game theory to improve our understanding of cancer dynamics. They study the interaction between malignant and normal cells in a multiple myeloma (MM) model (Dingli *et al*, 2009). I hope to give a simplified explanation of the underlying concepts to a non-mathematical physician, as well as pose some challenges to the authors.

Game theory has fascinating potential when applied to the field of medicine (Tarrant *et al*, 2004). At a fundamental level, the field of game theory evolved from the mathematical exploration of conflict situations between rational entities that make predictable and reproducible choices (Von Neumann and Morgenstern, 1944). Subsequent mathematical formulae are derived from and are dependent on these prerequisites.

The fact that both players make reproducible and rational choices allows for the prediction of equilibrium states in ‘games’ that are played repeatedly over time. This equilibrium state is the steady state in which the cumulative returns (payoff) of both ‘players’ from repeated interactions are maximised. In game theory, this payoff is known as a Util. There can be many possible equilibrium states for any one game; conversely, there is always at least one equilibrium state for any game that is finite and allows mixed strategies (alternating strategies are possible).

The idea of an equilibrium state has been successfully applied to evolutionary theory, most notably in the development of evolutionary stable strategies (ESSs) by George Price and John Maynard Smith (Maynard Smith, 1973). An ESS is essentially a strategy with a symmetric equilibrium state, except that it is also more stable than any possible alternative strategy to the game. This requirement provides the necessary evolutionary pressure against invasion from other competing strategies that would destabilise this equilibrium.

Another important concept in evolutionary game theory is that of ‘Fitness’. Fitness is commonly described as the average number of extra offspring that carry a specific trait (gene, replicator) into the next generation as a result of the trait being used in the current generation. The proportion of extra offspring increases at a rate proportional to the fraction of the population currently hosting the trait and also to the difference in fitness between the trait in question and the average fitness of all other competing traits in the population. In terms of the game, traits can be seen as strategies of play and fitness as the payoff (Utils).

In cancer dynamics, such ESSs are attractive heuristics in that they can theoretically be used to understand and thus potentially manipulate the process of cancer growth. One can potentially predict and effect improved survival by changing the strategies and payoffs (fitness) of the cancer ‘game’. This innovative paper attempts to do just that. The conclusion reached is that by reducing the fitness of malignant cells (their payoff) compared with the fitness of normal cells, one can potentially eradicate the cancer cell by natural selection.

Some basic game theory examples are useful in exploring this concept of reduced fitness. A classic example of a game model for evolution is the ‘Hawk–Dove Prisoners Dilemma Game’. In this game, two birds drawn from the same species compete for a valuable resource. Two traits in the population make their host either passive (a dove) or aggressive (a hawk). A dove surrenders the entire resource to a hawk, two doves share the resource equally, and two hawks fight (which has reduced fitness owing to the dangers of fighting).

This game has logical applicability to cancer dynamics in that the aggressive malignant cell (hawk) competes with a passive normal cell (dove) for biological energy. We can numerically express such strategies by their fitness (Utils). Two interacting dove cells have a hypothetical fitness of 2 Utils each (sharing), 1 Util each if both are hawks (fighting), and 4 to 0 Utils if one player is a hawk and the other is a dove, respectively. The resulting game theory ESS for this interaction is that in which all players become hawks, which seems contradictory as they would be more fit if they were both doves (Figure 1). This apparent contradiction is a fundamental concept in game theory. Thus, the appearance of even a tiny fraction of hawks dooms the doves to extinction. A similar result was seen by Dingli *et al* when they chose a value of *β*>1 for the fitness of the interaction between a myeloma cell (MM) and an osteoclast (OC). They found that the normal equilibrium was then disturbed by introducing one MM cell into a population of 10^10^ normal cells (see their Figure 2 (Dingli *et al*, 2009)). These scenarios resemble the end result of untreated cancer, in which malignant cells overcome normal cells. Therefore, backward induction suggests that this is a reasonable mathematical model for cancer dynamics.

The hypothesised conclusion of the Dingli paper suggests attempting to reduce the fitness of malignant cells (hawks). This game scenario is already modelled in evolutionary game theory as the ‘Hawk–Dove Chicken’ game. Here, the payoff for being a hawk is less, as even a slight injury from fighting is likely to be a severe handicap, leading to less fitness. The payoffs in this game are thus 2 Utils each if both are doves, −1 Util each if both are hawks, and 4 to 0 Utils if a hawk plays a dove. We have decreased the fitness of two hawks interacting by 2 Utils each. Such a decrease in fitness yields a different ESS in which ⅓ of the cells are normal (dove) and ⅔ are malignant (hawk) (Figure 2). In the context of Dingli *et al*'s paper, such a reduction could potentially be affected by therapies reducing *β*. This example also underscores the importance of targeted strategies that reduce the fitness of malignant cells alone, without any effect on the fitness of normal cells. New biological therapies have this potential and have been leading to improved outcomes (Weisberg *et al*, 2006).

It is clear that such a simplified approach can be developed further and be used to better understand the dynamics of malignant cell interactions with normal cells. Dingli *et al* attempt to demonstrate a possible eradication of MM cells by reducing the fitness of the interaction between OCs and MM cells (*β*), such that this was less fit than the interaction between osteoblasts (OBs) and OC (see their Figure 3 (Dingli *et al*, 2009)). They also demonstrate that altering the fitness numbers for the interaction between OBs and MM cells (*δ*) will affect the time needed to reach the new equilibrium between OCs and MM cells, and is thus important in prognosis (see their Figure 2D (Dingli *et al*, 2009)). These ideas have clear merit.

However, game theory has a number of problems when applied to oncology. From a prediction perspective, the actual ratio of normal to malignant cells that evolves in treatment-driven ESS depends on the fitness numbers artificially incorporated into the game for any particular strategy. It is therefore purely theoretical. It may be possible to determine the effect of a therapy on malignant cell fitness by extrapolating backwards from the observed ratio of malignant cells to normal cells after treatment; however, this is far beyond our current technologies.

Furthermore, whether a cancer cell is a ‘game player’ that can ever make rational choices leading to an equilibrium state (or ESS) is unclear. Immortalised cells develop and do not interact ‘rationally’ with normal cells to maximise the combined payoff. Their behaviour is in no way similar to that of rational evolutionary behaviour. They pass on mutated genes to mostly clonal offspring until all of the desired resource is consumed and they then die along with their host. This lack of rational behaviour seems to fundamentally limit their applicability to game theory mathematics.

Moreover, the idea of reducing the fitness of malignant cells is essentially an attempt to reverse the processes that made them cancer cells in the first place. Cancer cells can be divided into proliferative cells with a deregulated increase in cell turnover, or into antiapoptotic cells that are resistant to cell death. *A priori*, both situations lead to increased fitness over normal cells. Any ESS reached by treatment can only at best achieve an ESS in which normal cells coexist with malignant cells as seen in the ‘Hawk–Dove Chicken Game’. This ESS will always destabilise once new mutations develop in malignant cells over time, which increases their fitness over normal cells.

Therefore, it seems that the only way for normal cells to win the deadly ‘game’ of cancer is to exclude malignant cells from the game altogether! Further work by researchers such as Dingli *et al* is needed to resolve some of these current paradoxes.

## Figures and Tables

**Figure 1:**
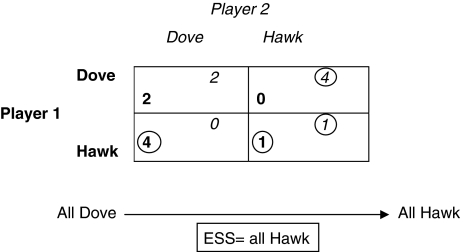
Prisoners' dilemma. The circled number is the fittest strategy for a given interaction. The interaction in which both strategies are circled is the equilibrium state (or in evolutionary parlance, the ESS). In this case, there is one best strategy (a pure ESS) and it is hawk–hawk. The strategy choices for player 1 are in bold and those for player 2 are in italics.

**Figure 2:**
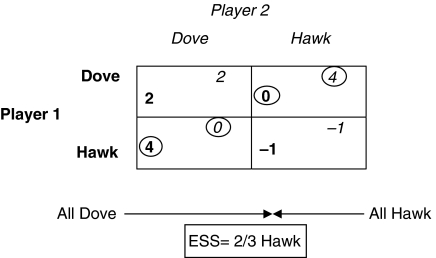
Chicken. In this case, there are two best strategies (a mixed ESS). The equilibrium is reached when ⅔ are hawk and ⅓ are dove. This ratio is a consequence of the numbers used for fitness in the model and is hypothetical but informative. The strategy choices for player 1 are in bold and those for player 2 are in italics.
